# Cytokine Profiling in Chagas Disease: Towards Understanding the Association with Infecting *Trypanosoma cruzi* Discrete Typing Units (A BENEFIT TRIAL Sub-Study)

**DOI:** 10.1371/journal.pone.0091154

**Published:** 2014-03-07

**Authors:** Cristina Poveda, Manuel Fresno, Núria Gironès, Olindo A. Martins-Filho, Juan David Ramírez, Julien Santi-Rocca, José A. Marin-Neto, Carlos A. Morillo, Fernando Rosas, Felipe Guhl

**Affiliations:** 1 Centro de investigaciones en Microbiología y Parasitología Tropical (CIMPAT), Facultad de Ciencias, Universidad de los Andes, Bogotá, Colombia; 2 Centro de Biología Molecular Severo Ochoa, Consejo Superior de Investigaciones Científicas (CSIC), Universidad Autónoma de Madrid (UAM), Cantoblanco, Madrid, Spain; 3 Laboratory of Diagnostic and Monitoring Biomarkers, Centro de Pesquisas René Rachou, Fundação Oswaldo Cruz - FIOCRUZ, Belo Horizonte, MG, Brazil; 4 Cardiology Division, Internal Medicine Department, Medical School of Ribeirao Preto, Universidad de Sao Paulo, Sao Paulo, Brazil; 5 Department of Medicine, Cardiology Division, McMaster University, PHRI-HHSC, Hamilton, Ontario, Canada; 6 Electrofisiología, Clínica Abood Shaio, Bogotá, Colombia; University of California Los Angeles, United States of America

## Abstract

**Background:**

Chagas disease caused by the protozoan *Trypanosoma cruzi* is an important public health problem in Latin America. The immunological mechanisms involved in Chagas disease pathogenesis remain incompletely elucidated. The aim of this study was to explore cytokine profiles and their possible association to the infecting DTU and the pathogenesis of Chagas disease.

**Methods:**

109 sero-positive *T. cruzi* patients and 21 negative controls from Bolivia and Colombia, were included. Flow cytometry assays for 13 cytokines were conducted on human sera. Patients were divided into two groups: in one we compared the quantification of cytokines between patients with and without chronic cardiomyopathy; in second group we compared the levels of cytokines and the genetic variability of *T. cruzi.*

**Results:**

Significant difference in anti-inflammatory and pro-inflammatory cytokines profiles was observed between the two groups cardiac and non-cardiac. Moreover, serum levels of IFN-γ, IL-12, IL-22 and IL-10 presented an association with the genetic variability of *T.cruzi*, with significant differences in TcI and mixed infections TcI/TcII.

**Conclusion:**

Expression of anti-inflammatory and pro-inflammatory cytokines may play a relevant role in determining the clinical presentation of chronic patients with Chagas disease and suggests the occurrence of specific immune responses, probably associated to different *T. cruzi* DTUs.

## Introduction

Chagas disease is considered by the World Health Organization (WHO) as one of the most important public health problems in Latin America, with an estimate of 7 million people infected and 110 million people at risk of contracting the disease [1], [2]. Recent evidence has also indicated that Chagas disease has migrated to non-endemic regions posing a significant social threat [3]. This parasitic disease is caused by the protozoan *Trypanosoma cruzi,* transmitted by different routes: via the feces of infected triatomine bugs, contaminated blood transfusion, oral (contaminated food/juices), vertical transmission, organs transplantation and laboratory accidents. Approximately 60% of patients in the chronic phase are clinically asymptomatic, around 30% manifest cardiomyopathy, 8% megavisceral syndromes and the remaining 2% both cardiomyopathy and digestive involvement [4], [5], [6], [7].

Two main hypotheses regarding the pathogenesis of Chagas disease have been proposed. One based on parasite persistence, and a second hypothesis based on adverse human host immune response to the infection, causing autoimmune aggression [8]. In the acute phase of the disease the host is able to control the infection in three different but complementary ways: i) Detection and direct destruction of the parasite by cells such as macrophages and dendritic cells, ii) activation of dendritic cells and macrophages that present antigen stimulation for the activation of the antigen-specific immune responses and iii) detection of infection by non-hematopoietic cells, which are the principal targets of the invasion by *T. cruzi* [9]. In the chronic phase, antigens that are elicited by the immune response are mediated by T cells, which play an important role in the progression of the disease. Studies in children with the indeterminate form have shown a high frequency of pro-inflammatory monocytes and regulatory cells compared with healthy subjects [10]. Thus, it is believed that expression of cytokines and their kinetics are important key factors influencing the development and progression of the disease. In fact, the balance between excessive pro-inflammatory cytokines and anti-inflammatory cytokines may be critical in the development of the chronic phase of the disease [5], [8], [11]. Studies on the specific role of cytokines in the immune response against *T. cruzi* have been recently reported and demonstrate that large amounts of Th1 pro-inflammatory cytokines such as Interferon Gamma (IFN-γ), Tumor Necrosis Factor Alpha (TNF-α) are related to cardiac disease [12]. It is also known that these cytokines are regulated by anti-inflammatory cytokines in low concentrations such as interleukin 10 and 5 (IL-10 and IL-5) [13]. Altogether, these data suggest a link between parasite infection, immune response polarization, and specific organ damage. Thus, cytokines may be used as biomarkers in order to identify the immune factors that influence disease progression has been carried out in different pathologies such as malaria, yellow fever, HIV infection, and also in Chagas disease [14], [15], [16]. It is believed that in these infectious diseases deregulation of host inflammatory responses play an important role and the profile of cytokines may be used as biomarkers of disease progression. Consequently, the characterization of these profiles may lead to future selection of treatment strategies targeting specific inflammatory pathways [17].

Several investigators have attempted to associate *T. cruzi* genetic variability and the different clinical manifestations of Chagas disease. The genetic variability within the *T. cruzi* taxon has been demonstrated using biological, biochemical and molecular markers, and has been grouped into six Discrete Typing Units (DTUs), TcI-TcVI [18], [19]. TcI is mostly related to the sylvatic cycle in the Amazon region with sporadic cases in the domestic foci recently named TcI_DOM_ [20]. However, TcI has recently gained relevance since it has been associated with severe cardiomyopathic manifestations in patients from Argentina and Colombia [19], [21], [22], [23]. TcII is the principal agent of Chagas disease in the Southern Cone of America and *Triatomainfestans* is the principal vector. TcV and TcVI, are derived from the hybridization between TcIII and TcII/TcIV and the cycle of transmission is mainly domestic [18], [24]. These DTUs are related not only to heart disease but also to megavisceral syndromes [25], [26]. TcIII and TcIV are mainly related to the sylvatic cycle of transmission and scarce evidence of human infection has been reported [27], [28], [29].

The aim of this study was to evaluate the different cytokine profiles in Chagasic patients with and without chronic cardiomyopathy to correlate these cytokine profiles in patients infected with the different *T.cruzi*DTUs in order to establish whether these factors may be involved in the pathogenesis of Chagas disease. Our results show that specific anti-inflammatory and pro-inflammatory cytokine modulation is correlated with the clinical presentation of chronic patients with Chagas disease and suggests the occurrence of specific immune responses, probably associated to different *T. cruzi* DTUs.

## Results

### Signature Profiles

#### Group I: CARD vs. NON-CARD

**Figure 1:**
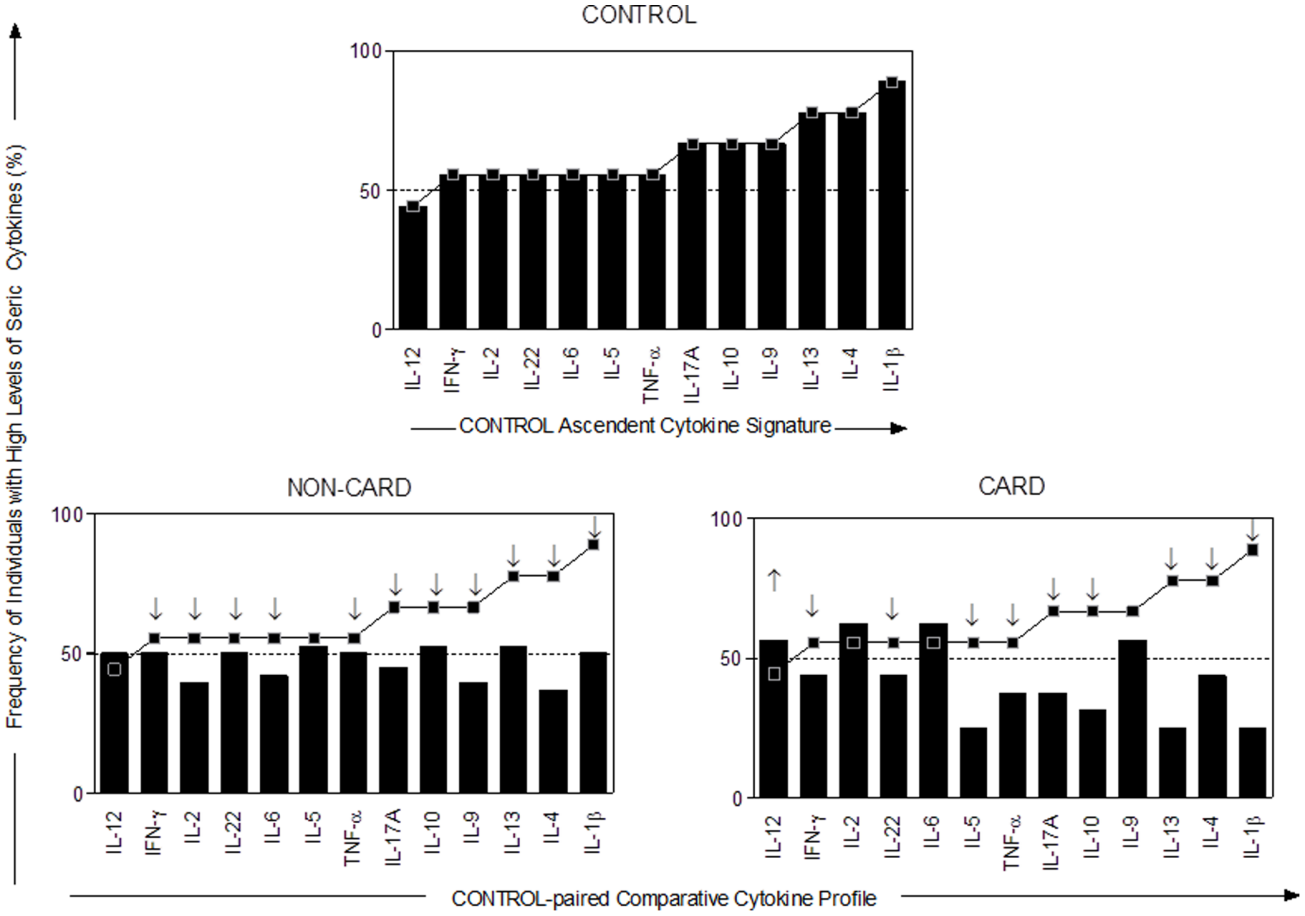
Cytokine Signatures with Frequency of Subjects with High Cytokine Levels. The diagrams were plotted using the global median of each MFI cytokine index as the cut-off mark to identify higher levels. The ascendant frequency of high cytokine producers at the with (CARD) and without Chagas cardiomyopathy (NON-CARD) was demonstrated by bar graphics. The ascendant frequency of high cytokine producers of the CONTROL group was used to generate the reference cytokine signature curves that were applied to identify changes in the overall cytokine signature from all other groups.

To identify possible markers of infection, and more specifically of cardiomyopathy, we measured seric cytokines in 16 patients with cardiomyopathy (CARD), 38 non-cardiac chagasic patients (NON-CARD), and 9 non-infected individuals (CONTROL). As expected in the case of immune responses against infection, cytokine production greatly varies among individuals, rendering inaccurate the use of the mean as central indicator. We thus used the mean of cytokine concentration, the latter being evaluated by the mean fluorescence intensity (MFI) of beads bound to cytokines recognized by immunofluorescence. The overall median of each individual MFI cytokine was calculated (IL-1β = 63.32, IL-2 = 49.28, IL-4 = 45.92, IL-5 = 58.24, IL-6 = 276, IL-9 = 181.4, IL-10 = 85.68, IL-12p70 = 54.6, IL-13 = 33.04, IL-17A = 44.8, IL-22 = 82.88, IFN-γ = 48.8 and TNF-α = 108.6) from patients of different clusters. These values were used as the cut-off mark to tag the cell population from each patient as being a high or low cytokine producer (Figure S1 in File S1). Then, in each group and for each cytokine, results were expressed as the frequency of individuals with a concentration of seric cytokine higher than the median of all samples (Figure 1). The assembling of the ascendant frequency of high cytokine producers for CONTROL generated the reference cytokine signature applied to identify changes in the overall cytokine signature from the CARD and NON-CARD groups. The results showed that the frequency of high cytokine producers in the CARD and NON-CARD groups was lower than in the CONTROL group.

**Figure 2:**
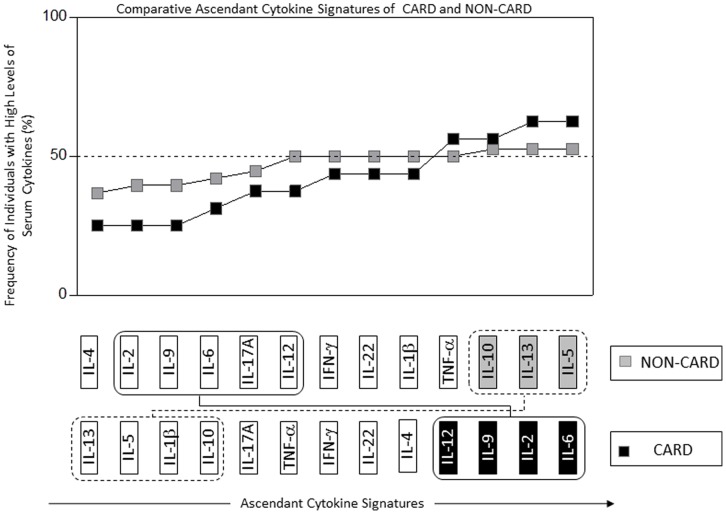
Comparative Cytokine Signatures of NON-CARD and CARD. The diagram is a comparison between the signing of the NON-CARD (▪) and CARD (▪). There is a switch between pro-inflammatory with the boxes in solid line (IL-6, IL-2, IL-9 and IL-12) and anti-inflammatory with the boxes in dashed line (IL-5,IL-13 and IL-10) cytokines; the CARD have higher frequencies of proinflammatory cytokines and lower levels of inflammatory cytokines, whereas the opposite occurs in the case of NON-CARD.

A switch among anti-inflammatory and pro-inflammatory cytokines profiles in the CARD and NON-CARD groups of patients was observed. The NON-CARD patients showed higher frequency of anti-inflammatory cytokines and lower frequency of pro-inflammatory cytokines while the CARD displayed higher pro-inflammatory and lower anti-inflammatory cytokines. The cytokines involved in the anti-inflammatory profile were IL-13, IL-5 and IL-10 and those in the pro-inflammatory profile were: IL-2, IL-6, IL-9 and IL12 (FIGURE 2).

#### Group II: *T. cruzi* variability

**Figure 3:**
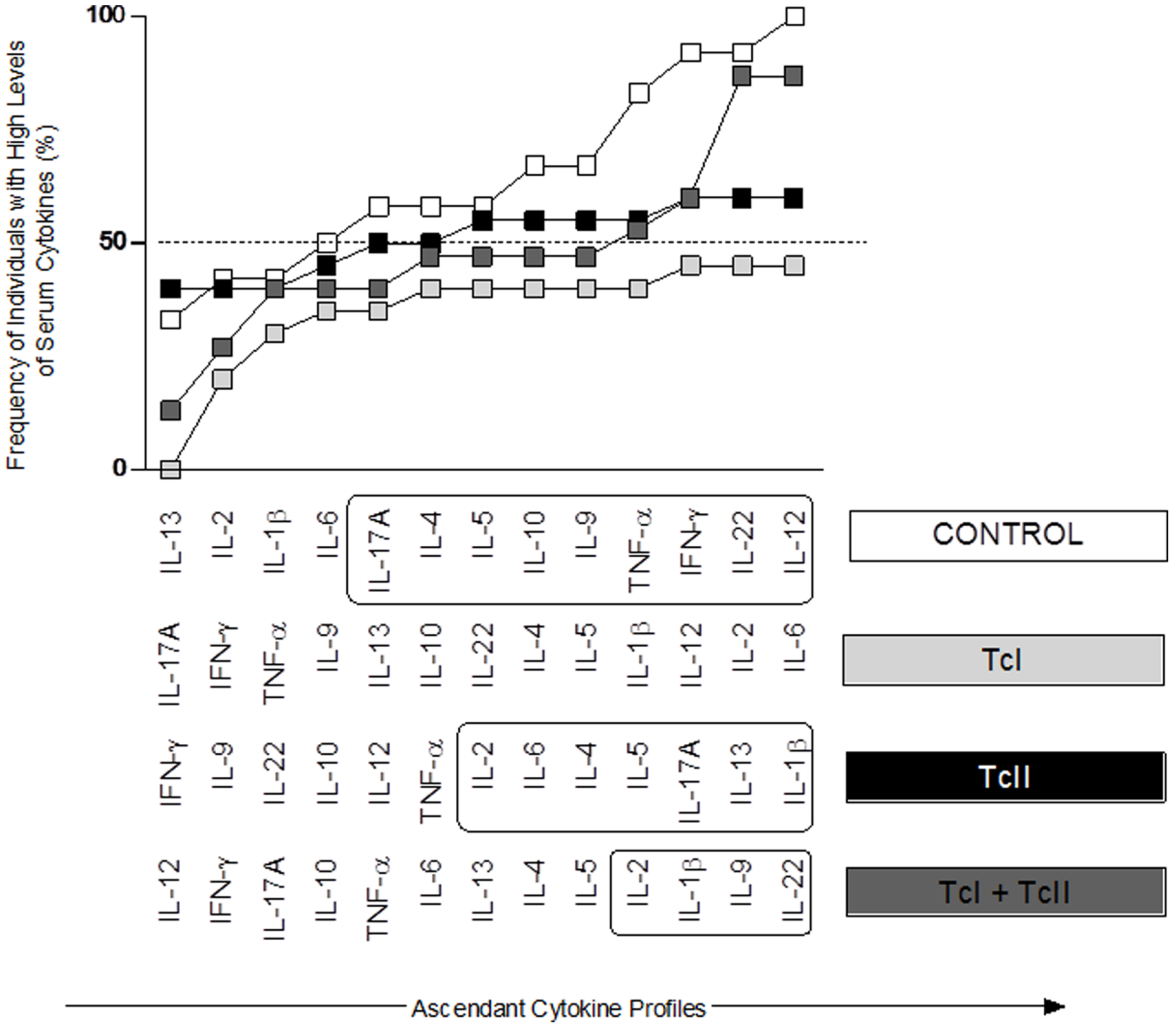
Comparison of Immunological Signatures between patients with chronic chagasic cardiomyopathy infected with different DTU’s. The diagram is a comparison between the signing of the TcI (▪), Tc II (▪), Mixed TcI+TcII (▪) and CONTROL (□). Cytokines with frequency equal or higher than 50% are in boxes.

As for Group I,overallmedian of each MFI cytokine wascalculated (IL-1β = 292,32, IL-2 = 224, IL-4 = 209.44, IL-5 = 251.44, IL-6 = 1197, IL-9 = 792.4, IL-10 = 369.6, IL-12p70 = 228.48, IL-13 = 160.16, IL-17A = 207.76, IL-22 = 547.12, IFN-γ = 198.24 and TNF-α = 465.92). These values were used as the cut-off mark to tag the cell population from each patient as being a high or low high cytokine producers(Figure S2 in File S1). Assembling of the ascendant frequency of high cytokine producers for CONTROL generated the reference cytokine signature applied to identify changes in the overall cytokine signature from all other groups: TcI, TcII and Mixed TcI/TcII (FIGURE 3).

A pro-inflammatory pattern was observed for all infected groups, but no particular pattern could be associated to the different DTUs. The frequency of cytokines in TcI, TcII and Mixed TcI/TcII showed lower frequency as compared with the CONTROL levels.

All the quantitative results provided by the cytometry system are included and can be found in Tables S1 and S2 in File S2.

### Statistical Analysis

This analysis aimed to establish associations between levels of cytokines and the clinical manifestation (CARD or IND) and/or the genetic variability of *T.cruzi* (TcI, TcII and Mixed TcI/TcII). Analysis of principal components was performed with the 13 cytokines studied. The principal components for the study group CARD vs NON-CARD were IL-12 = 0.990, IFN-γ = 0.990, IL-1 = 0.920, IL-6 = 0.915 and IL-9 = 0.895 which explained the model in 80.4% of the variation in the dataset; for the study group II *T. cruzi* DTUs the principal components were IFN-γ = 0.882, IL-12 = 0.822, IL-22 = 0.817 and IL-10 = 0.805 explaining 75.42% of variation (Tables S3 to S8 in File S2).

**Figure 4:**
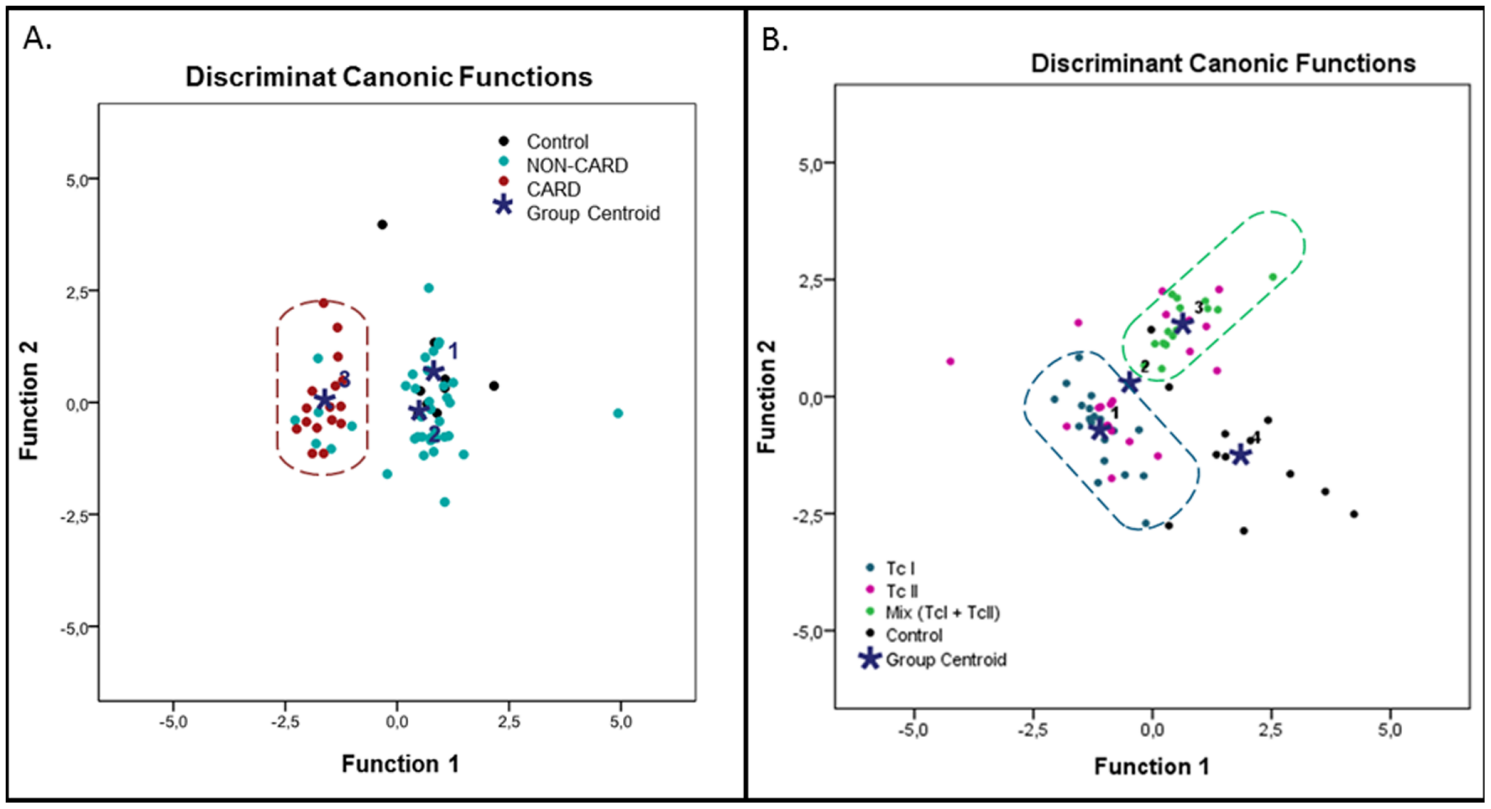
Discriminant Analysis of Principal Components (DAPC). 4A. DAPC for with and without cardiomyopathy Chagas patients and CONTROLS. The diagram show a cluster of 100% for CARD (Red circle), 55,3% for NON-CAR (Cian Circel) and 55,6% for the CONROL (•). 4B. DAPC of patients with chronic Chagas cardiomyopathy infected with different DTU’s and CONTROLS: The diagram shows a cluster of 95% for TcI (Blue Circle), 15% for Tc II (Magenta Circle), 86,7% for mix TcI/TcII (Green Circle) and 83,3% for the CONTROL (•).

Finally, discriminant analysis was performed to observe which proportion of the selected components were actually able to group the study groups; for study group I (CARD vs NON-CARD) the components group in the right cluster (when both real and theory group coincide) : NON-CARD = 21/38 (55.3%), CAR = 16/16 (100%) and CONTROL = 5/9 (55.6%) (FIGURE 4 a); and for the study group II (*T. cruzi* DTUs) the components group in the right cluster TcI = 19/20 (95%), TcII = 3/20(15%), Mixed TcI/II = 13/15(86,7%) and CONTROL = 10/12(83,3%) (FIGURE 4 b). These results show a clear profile in patients with CARD, and an association between levels of cytokines and the variability of *T.cruzi* (TcI, TcII and Mix TcI/TcII)(Tables S9 and S10 in File S2.).

## Discussion

An important finding of this study was a switch between the anti-inflammatory cytokines IL-13, IL-5 and IL-10 and pro-inflammatory cytokines IL-2, IL-6, IL-9 and IL-12 among CARD and NON-CARD groups. These findings suggest that regulation (differential regulation of cytokine synthesis) may play an important role in the development of Chagas cardiomyopathy.

The anti-inflammatory/pro-inflammatory cytokine profile switch found between patients with Chagas cardiomyopathy and those infected but still in the NON-CARD stage of the disease is compatible with the hypothesis that the progression of human Chagas disease from asymptomatic to severe forms, is related with a lack of adequate immune modulation [8]. While an appropriate, inflammatory, response may be beneficial in early stages of infection, the lack of control of this response later on the outcome of the disease will allow for the establishment of tissue damage. This hypothesis is in agreement with the finding that IL-10 levels were diminished in comparison to levels of TNF- α and IFN-γ in CARD patients, in contrast with the opposite profile (higher levels of IL-10 than levels of TNF- α and IFN-γ) in indeterminate form patients [8]. These findings are also in keeping with a recent report documenting increased matrix metalloproteinases 2 and 9 in *T. cruzi*seropositive subjects with and without Chagas cardiomyopathy manifestations [30]. However, previous reports have not documented that these pro-inflammatory cytokines play an important role in producing Chagas disease immunomodulation disequilibrium [8], [31], [32].

Pro-inflammatory cytokines involved in the switch were IL-2, IL-9, IL-6 and IL-12. The role of the last two cytokines has already been studied in Chagas disease. Thus, the presence of high levels of IL-6 in CARD patients was correlated with the severity of myocardium damage, in Peruvian and Colombian populations [33]. Another study determined the role of genetic polymorphism in the IL-12, and it was the C allele of IL-12B 3′UTR that acted as a risk factor and influenced the susceptibility to develop cardiomyopathy in Chagas disease [34]. Our results are in agreement with these studies and additionally document that these cytokines have higher levels in CARD patients compared to NON-CARD patients. On the other hand, the anti-inflammatory cytokines involved in the switch were IL-13, IL-5 and IL-10. IL-10 was the most important anti-inflammatory cytokine studied in the Chagas disease, and it has been shown that NON-CARD patients display higher levels of IL-10 in comparison to cardiac patients [8], [35]. Other investigators evaluated the SNP’s in indeterminate and CARD populations, and also divided the cardiac patients between those with non-dilated and dilated hearts, finding different allele and genotype frequencies among them; also, a correlation was found between the allele A of -1082G/A in the IL-10 and low levels in CARD patients [36].

On the other hand, the relationship of the genetic variability of the parasite and host characteristics with pathogenesis is increasingly recognized as an important factor in the understanding of Chagas disease. In all our groups independent of the genetic variability of the infecting *T.cruzi* DTUs a pro-inflammatory profile was observed. Moreover, all those groups manifested a pro-inflammatory profile similar to the one we found in the study group I (CARD and NON-CARD). However, the TcII and Mixed groups showed higher levels of cytokines compared to the TcI, where the levels did not exceed 50%. It is interesting to speculate that the lack of a highly specific profile associated to a particular infecting-DTU is likely due the pleiotropic and somewhat redundant role of many cytokines. For all DTUs groups the profile is pro-inflammatory, and probably this pro-inflammatory profile will eventually lead to the same clinical manifestation (cardiomyopathy), regardless of the different DTUs with which the patients were infected and of some particular cytokine responses which are DTU-dependent. Thus, we found higher levels of the cytokines IL-6 for TcI, IL-1 for TcII and of IL-22 for Mixed TcI/TcII. This is relevant, since it is known that IL-6 is secreted by T cells and macrophages, IL-1 by macrophages and lymphocytes, and IL-22 by dendritic cells and T cells [37].

Presumably, DTU-specific recognition by the immune system (Antibodies, B cells, or T cells), could lead to the differential responses observed. Thus the DTUs-differential recognition by host receptors, that recognized pathogen patterns and their cognate pathogen associated molecular patterns (PAMPs), could be linked with the observed different induction of pro-inflammatory cytokines. In this regard, previous reports, demonstrated that GPI-anchors from *T.cruzi* surface antigens triggered the synthesis of IL-12 by macrophages and dendritic cells through toll-like receptor 2 (TLR-2) dependent activation [38]. In addition, TLR-9 that recognizes T. cruziCpG-rich DNA also stimulated the production of IL-12, IFN-γ and TNF-α by macrophages. The glycoinositolphospholipid from epimastigotes of *T. cruzi* is recognized as TLR-4 in which also trigger IL-12, IL-2 and TNF. In addition, other cells, known to be infected *T. cruzi,* as myocytes and adipocytes, may contribute to the cytokine profile observed. Since IL-6 is also produced by adipocytes [9].

The DAPC analysis showed a clear grouping for the CARD patients but not for NON-CARD patients. This result may be due to the fact that some patients in the NON-CARD group could be eventually more predisposed to develop cardiomyopathy or mega-viscera syndrome explaining why cytokine levels are not homogeneous for this group. In the study group, the DAPC analysis suggests that there is association between the genetic variability of *T. cruzi* (Tc I, Tc II and Mixed TcI/TcII) and the levels of some cytokines. We found again in both groups, I and II, that IL-12, IFN-γ, IL-6 and IL-1 are the principal components and thus they may play an important role in Chagas disease pathophysiology. Also, differences in the frequency of high producers of IL-17 among in patients infected with different DTUs were found: 0% for TcI, 60% for TcII and 40% for mixed infection (TcI/TcII). Those differences may lead to slightly different outcomes of the disease. In this regard, previous studies showed that patients with less aggressive cardiomyopathy forms of the disease produce higher levels of IL-17 [39], suggesting that in our study the patients with more severe cardiomyopathy infection would be those with TcI followed by those with mixed infection and finally those infected with TcII.

In summary, pro-inflammatory profile is associated to CARD patients and anti-inflammatory profile to NON-CARD patients. The fact that the levels of cytokines were different for patients infected with different parasite DTUs suggests a specific immune response, probably associated to DTUs. A novel contribution of this study is the possibility of using IL-12, IFN-γ, IL-6 and IL-1 as prognostic biomarkers of Chagas Disease. Future studies should be carried out covering the geographical wide-range of DTUs and other human populations, in order to validate these cytokines as biomarkers as well as finding the plausible association between DTUs-epitope and Chagas Disease clinical manifestations.

## Materials and Methods

### Human Sera

Following WHO criteria for Chagas disease diagnosis, two serological tests (ELISA and IIF) were employed to enroll the patients. According to these diagnostic test results, a total of 109 serum samples from Chagas patients and 21 serum samples from healthy donors were assayed, aged 40 to 50 years living in rural areas of Colombia and Bolivia (77 individuals from Santander, Colombia and 53 from Cochabamba and Tarija, Bolivia). Chagas patients were classified as without cardiomyopathy (NON-CARD  = 38) when they were seropositive with no evidence of cardiac disease (based on the absence of symptoms, physical signs and of normal X-ray, electrocardiogram and/or echocardiogram). Patients with chronic Chagas cardiomyopathy (CARD = 71) were characterized according to clinical criteria and abnormalities in chest X-ray, electrocardiogram and/or echocardiogram. The presence of other acute, hepatic, renal or psychiatric diseases were a motive for exclusion criteria.

The studies were conducted in two groups of individuals: the first one to compare the differences in the quantification of cytokines in patients with and without chronic cardiomyopathy and control individuals; and the second one to compare the differences between the levels of cytokines and the genetic variability of *T. cruzi.*

Group I (CARD and NON-CARD), included 54 serum samples from Chagas patients, 38 being classified as NON-CARD and 16 patients as CARD, and 9 serum samples from CONTROL individuals. The selection criteria of the patients groups were trying based on the assumption of obtaining the percentage to simulate the % of Chronic Chagasic manifestation reports 38/54 chagasicpatientes (70.37% NON-CARD) against not Chronic Chagasicmanifestationnd 16/54 (29.62% CARD).

Group II (*T. cruzi* DTUs), included 55 serum samples from Chagas patients with chronic cardiomyopathy and 12 CONTROL individuals. Twenty patients were typed as TcI, 20 as TcII and 15 mixed TcI/II. These sera were previously genotyped by Ramirez *et al.* 2010, where the initial sample size was 240 patients. Only 20/240 patients were genotyped as Mixed infection being TcI+ TcII (5/20 sera were contaminated with bacteria and subsequently excluded)we use the same sample size of patients for the others DTUs for optimal statistical analyzes.

#### Experimental ethics

Written and oral consent was obtained in all patients included as part of the BENEFIT trial (Benznidazole Evaluation for Interrupting Trypanosomiasis), the study is approved by all local and national Institutional Review Board (ClinicaAboodShaio, Hospital de la Policia, Fundacion Cardiovascular de San Gil Santander), the study is approved by the Ethics Research Committe of the World Health Organization as one of the funding agencies of the BENEFIT trial.

### Cytokine Quantification and Flow Cytometry Acquisition

Fluorescent bead-based flow cytometry assays for 13 anti-inflammatory and pro-inflammatory cytokines was performed by duplicate (Human Th1/Th2/Th9/Th17/Th22 13plex FlowCytomix Multiplex, Bender MedSystems GmbH, eBioscience), on human sera (without *in-vitro* stimulation), following the manufacturer’s protocol. Briefly, combining 2 different bead sizes and 6 or 7 fluorochrome densities for each population (thus fluorescence intensities, with an emission maximum in the far red range), 13 different bead populations are defined, each one associated to a specific antibody. Cytokines are trapped by these antibody-bead complexes, and then recognized by another antibody in solution; the latter is previously bound to a secondary antibody coupled to phycoerythrin (PE).

Sample reading was performed in BIO FACS Canto II™ Becton Dickinson (BD) cytometer and analyzed with eFlowCytomix Pro software (Cat. No. BMS8402FF). After acquiring 30,000 events/microwell, cytokine concentration was analyzed according to manufacturer’s recommendation. After gating the two bead sizes on a dot plot with forward scatter (FSC) and side scatter (SSC), events were plotted according to fluorescence emission at 700 nm (bead-contained fluorochrome, intensity corresponding to cytokine identity) and at 595 nm (PE emission peak, intensity corresponding to cytokine concentration). Fluorescence intensity will further refer to the intensity recorded at 595 nm.

### Signatures Analyses

The cytokine profile was first assessed by identifying low and high cytokine producers, as previously reported [40]. Briefly, after the establishment of the overall median of Median of Fluorecence Intensity (MFI), each cytokine subsets from all sub-groups were tagged as they displayed low or high cytokine production. The percentage of sub-groups showing high cytokine indexes was calculated for each one. The ascendant frequency of high cytokine indexes of controls was then used as the reference cytokine curves to identify changes in the overall cytokine patterns from all other sub-groups. Each axis represents the frequency (%) of volunteers showing high cytokine indexes.

### Statistical Analysis

Statistical analyses of the data were carried out using GraphPad Prism software 5.0 and Statistical Package for the Social Sciences 17, SPSS. Discriminant Analysis of Principal Components (DAPC) was performed, to assess the association between the different cytokines and the clinical manifestation of Chagas disease (CARD or NON-CARD) and/or the variability of *T.cruzi* (TcI, TcII and Mixed TcI/TcII). The totals of components considered were able to explain up to 80% of the model. The analysis of cytokine signatures was performed using the control cytokine signature as the reference curve, and significant differences were considered when the values emerged outside the quartile of the reference signature.

## Supporting Information

File S1 **File S1 includes the following: Figure S1. Ilustrative diagram of Chagasic patientes with and without chronic cardiomyopathy and control.** The diagrams were plotted using the global median cytokine index as the cut-off mark to identify as a low (□) or high (▪) cytokine producer. **Figure S2. Ilustrative diagram of patients with chronic Chagas cardiomyopathy infected with different DTU**’**s and control.** The diagrams were plotted using the global median cytokine index as the cut-off mark to identify as a low (□) or high (▪) cytokine producer.
(PDF)

File S2 **File S2 includes the following: Table S1.** MFI of with (CARD) and without (NON-CARD) chronic cardiomyopathy chagas patients and control. **Table S2**. MFI of patients with chronic Chagas cardiomyopathy infected with different DTU’s and control **Table S3.** Kaiser-Meyer**-**Olkin and Barlett’s Test for with and without chronic cardiomyopathy chagasic patients and control. **Table S4.** Communalities for with and without chronic cardiomyopathy chagasic patients and control. **Table S5**. Total Variance Explanied for with and without chronic cardiomyopathy chagasic patients and control. **Table S6.** Kaiser-Meyer**-**Olkin and Barlett’s Test for patients with chronic Chagas cardiomyopathy infected with different DTU’s and control. **Table S7.** Communalities for patients with chronic Chagas cardiomyopathy infected with different DTU’s and control. **Table S8.** Total Variance Explanied for patients with chronic Chagas cardiomyopathy infected with different DTU’s and control. **Table S9.** Classification Result for discriminant analyze for with and without chronic cardiomyopathy chagasic patients and control. **Table S10.** Classification Result for discriminant analyze for patients with chronic Chagas cardiomyopathy infected with different DTU’s and control.
(DOCX)
