# Supplementary material for: Cytokine Profiling in Chagas Disease: Towards Understanding the Association with Infecting Trypanosoma cruzi Discrete Typing Units (A BENEFIT TRIAL Sub-Study)
Source: PLoS One. 2014 Mar 7;9(3):e91154. doi: 10.1371/journal.pone.0091154 (PMC3946691; doi:10.1371/journal.pone.0091154)
Supplement: File S1 — includes the following: Figure S1. Ilustrative diagram of Chagasic patientes with and without chronic cardiomyopathy and control. The diagrams were plotted using the global median cytokine index as the cut-off mark to identify as a low (□) or high (▪) cytokine producer. Figure S2. Ilustrative diagram of patients with chronic Chagas cardiomyopathy infected with different DTU’s and control. The diagrams were plotted using the global median cytokine index as the cut-off mark to identify as a low (□) or high (▪) cytokine producer. (PDF) [file pone.0091154.s001.pdf]

FileS1

Control

Seric Cytokines

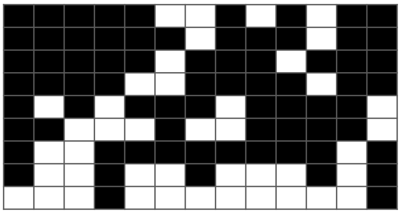

	IL-1β	IL-6	TNF-α	IL-4	IL-5	IL-12	IFN-γ	IL-2	IL-9	IL-17A	IL-22	IL-10	IL-13
Total	9	9	9	9	9	9	9	9	9	9	9	9	9
N º High Producers	8	5	5	7	5	4	5	5	6	6	5	6	7
%	89	56	56	78	56	44	56	56	67	67	56	67	78

CARD

Seric Cytokines

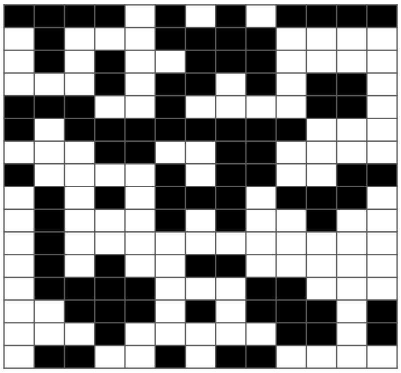

	IL-1β	IL-6	TNF-α	IL-4	IL-5	IL-12	IFN-γ	IL-2	IL-9	IL-17A	IL-22	IL-10	IL-13
Total	16	16	16	16	16	16	16	16	16	16	16	16	16
N º High Producers	4	10	6	7	4	9	7	10	9	6	7	5	4
%	25	63	38	44	25	56	44	63	56	38	44	31	25

NON-CARD

Seric Cytokines

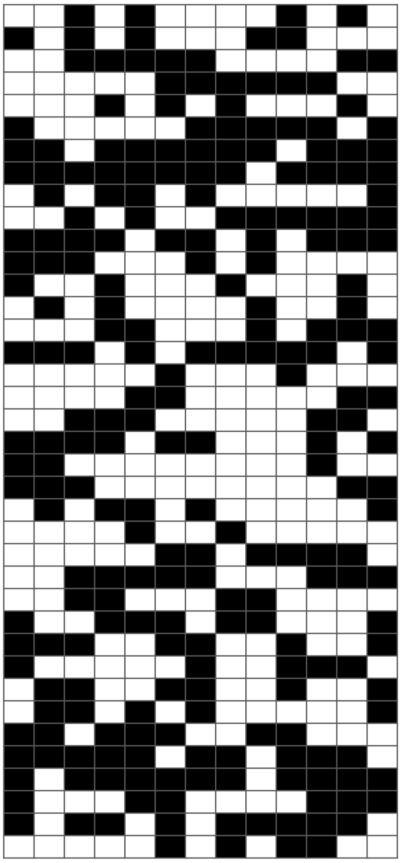

	IL-1β	IL-6	TNF-α	IL-4	IL-5	IL-12	IFN-γ	IL-2	IL-9	IL-17A	IL-22	IL-10	IL-13
Total	38	38	38	38	38	38	38	38	38	38	38	38	38
N º High Producers	19	16	19	14	20	19	19	15	15	17	19	20	20
%	50	42	50	37	53	50	50	39	39	45	50	53	53

Figure S1

Tc I

Seric Cytokines

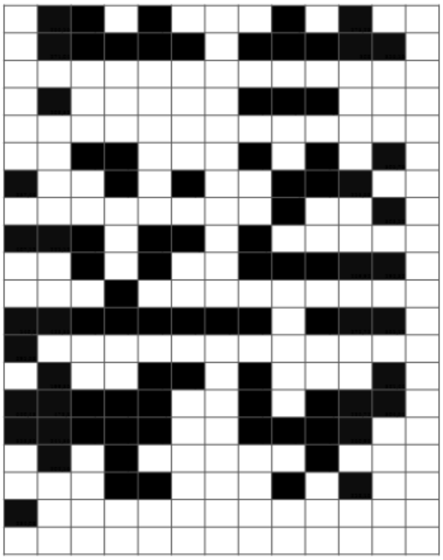

	IL-12	IFN-γ	IL-17A	IL-2	IL-10	IL-9	IL-22	IL-6	IL-13	IL-4	IL-5	IL-1	TNF-α
TOTAL	20	20	20	20	20	20	20	20	20	20	20	20	20
Nº High Level	7	9	8	8	9	4	0	9	8	7	8	6	0
%	35	45	40	40	45	20	0	45	40	35	40	30	0

Tc II

Seric Cytokines

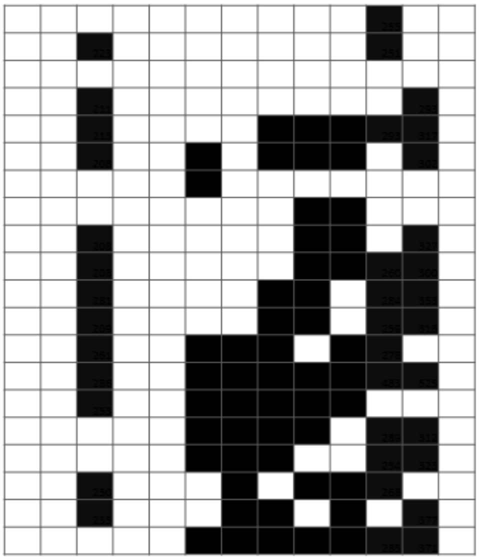

	IL-12	IFN-γ	IL-17A	IL-2	IL-10	IL-9	IL-22	IL-6	IL-13	IL-4	IL-5	IL-1	TNF-α
TOTAL	20	20	20	20	20	20	20	20	20	20	20	20	20
Nº High Level	0	0	12	0	0	8	8	11	12	11	11	12	0
%	0	0	60	0	0	40	40	55	60	55	55	60	0

Mixed Tc I/II

Seric Cytokines

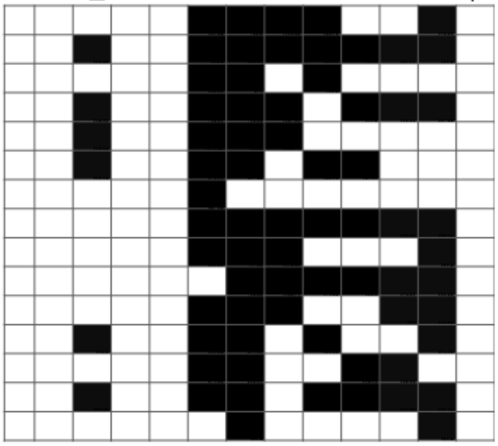

	IL-12	IFN-γ	IL-17A	IL-2	IL-10	IL-9	IL-22	IL-6	IL-13	IL-4	IL-5	IL-1	TNF-α
TOTAL	15	15	15	15	15	15	15	15	15	15	15	15	15
Nº High Level	0	0	6	0	0	13	13	7	7	7	7	9	0
%	0	0	40	0	0	87	87	47	47	47	47	60	0

CONTROL

Seric Cytokines

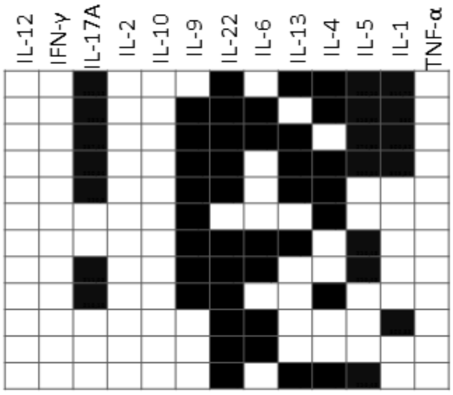

	IL-12	IFN-γ	IL-17A	IL-2	IL-10	IL-9	IL-22	IL-6	IL-13	IL-4	IL-5	IL-1	TNF-α
TOTAL	12	12	12	12	12	12	12	12	12	12	12	12	12
Nº High Level	0	0	7	0	0	8	11	6	4	7	7	5	0
%	0	0	58	0	0	67	92	50	33	58	58	42	0

Figure S2
