# Supplementary material for: Cytokine Profiling in Chagas Disease: Towards Understanding the Association with Infecting Trypanosoma cruzi Discrete Typing Units (A BENEFIT TRIAL Sub-Study)
Source: PLoS One. 2014 Mar 7;9(3):e91154. doi: 10.1371/journal.pone.0091154 (PMC3946691; doi:10.1371/journal.pone.0091154)
Supplement: File S2 — includes the following: Table S1. MFI of with (CARD) and without (NON-CARD) chronic cardiomyopathy chagas patients and control. Table S2. MFI of patients with chronic Chagas cardiomyopathy infected with different DTU’s and control Table S3. Kaiser-Meyer-Olkin and Barlett’s Test for with and without chronic cardiomyopathy chagasic patients and control. Table S4. Communalities for with and without chronic cardiomyopathy chagasic patients and control. Table S5. Total Variance Explanied for with and without chronic cardiomyopathy chagasic patients and control. Table S6. Kaiser-Meyer-Olkin and Barlett’s Test for patients with chronic Chagas cardiomyopathy infected with different DTU’s and control. Table S7. Communalities for patients with chronic Chagas cardiomyopathy infected with different DTU’s and control. Table S8. Total Variance Explanied for patients with chronic Chagas cardiomyopathy infected with different DTU’s and control. Table S9. Classification Result for discriminant analyze for with and without chronic cardiomyopathy chagasic patients and control. Table S10. Classification Result for discriminant analyze for patients with chronic Chagas cardiomyopathy infected with different DTU’s and control. (DOCX) [file pone.0091154.s002.docx]

**File S2.**

**Table S1.** MFI of with (CARD) and without (NON-CARD) chronic cardiomyopathy chagas patients and control.

| Group. | IL-12 | INF-γ | IL-17 | IL-2 | IL-10 | IL-9 | IL-22 | IL-6 | IL-13 | IL-4 | IL-5 | IL-1β | TNF-α |
| --- | --- | --- | --- | --- | --- | --- | --- | --- | --- | --- | --- | --- | --- |
| CONTROL | 50,4 | 37,52 | 56 | 60,48 | 89,6 | 180,32 | 80,08 | 277,2 | 40,32 | 52,64 | 63,84 | 70,56 | 109,76 |
|  | 64,96 | 41,44 | 57,68 | 56,56 | 104,72 | 187,04 | 82,88 | 302,4 | 43,68 | 52,64 | 63,84 | 81,2 | 282,24 |
|  | 45,92 | 49,28 | 43,68 | 53,76 | 87,36 | 183,68 | 88,48 | 421,12 | 36,96 | 54,88 | 64,96 | 76,16 | 145,6 |
|  | 49,84 | 53,76 | 48,72 | 51,52 | 86,24 | 196,56 | 75,6 | 284,48 | 34,16 | 49,28 | 58,24 | 81,76 | 123,2 |
|  | 75,6 | 56 | 45,92 | 48,16 | 89,04 | 183,12 | 92,4 | 273,28 | 30,24 | 44,8 | 68,32 | 71,68 | 114,24 |
|  | 66,08 | 36,96 | 66,64 | 46,48 | 94,08 | 235,76 | 83,44 | 284,48 | 32,48 | 40,32 | 58,24 | 78,4 | 107,52 |
|  | 70,56 | 63,84 | 57,12 | 54,32 | 81,76 | 183,68 | 92,4 | 247,52 | 42,56 | 49,28 | 66,08 | 77,28 | 103,6 |
|  | 50,4 | 50,96 | 40,32 | 47,6 | 85,68 | 180,32 | 97,44 | 262,64 | 42,56 | 58,24 | 54,88 | 82,88 | 108,64 |
|  | 52,08 | 43,68 | 36,96 | 48,16 | 85,12 | 178,08 | 81,76 | 275,52 | 35,84 | 47,04 | 52,64 | 62,72 | 108,64 |
| NON-CARDIAC | 42,56 | 41,44 | 50,4 | 42 | 90,72 | 171,92 | 77,28 | 268,8 | 32,48 | 44,8 | 62,16 | 67,2 | 112 |
|  | 41,44 | 34,16 | 45,92 | 43,68 | 84 | 189,28 | 79,52 | 272,16 | 28,56 | 44,8 | 61,6 | 71,68 | 111,44 |
|  | 2806,72 | 329,84 | 44,8 | 43,12 | 87,36 | 169,12 | 76,72 | 268,8 | 36,4 | 47,04 | 59,36 | 68,32 | 109,76 |
|  | 60,48 | 52,64 | 56 | 58,24 | 76,72 | 211,68 | 84 | 267,68 | 30,24 | 39,2 | 50,4 | 67,2 | 106,96 |
|  | 70,56 | 37,52 | 41,44 | 50,4 | 90,72 | 174,16 | 77,28 | 264,32 | 33,04 | 45,92 | 58,24 | 63,84 | 105,28 |
|  | 41,44 | 51,52 | 56 | 56 | 75,04 | 193,76 | 87,36 | 262,08 | 33,6 | 43,68 | 58,24 | 71,12 | 107,52 |
|  | 160,16 | 64,96 | 43,68 | 59,36 | 104,72 | 184,8 | 100,8 | 290,64 | 39,76 | 58,8 | 66,08 | 80,64 | 108,08 |
|  | 63,84 | 48,72 | 81,76 | 92,96 | 115,36 | 166,32 | 113,12 | 279,44 | 35,84 | 104,72 | 62,16 | 135,52 | 156,8 |
|  | 52,64 | 45,92 | 39,2 | 44,8 | 78,96 | 170,8 | 78,4 | 285,04 | 35,84 | 45,92 | 63,84 | 62,72 | 106,4 |
|  | 47,04 | 44,8 | 48,72 | 51,52 | 89,04 | 193,76 | 96,32 | 263,2 | 34,72 | 43,68 | 60,48 | 64,96 | 112 |
|  | 73,36 | 62,72 | 44,8 | 42,56 | 87,92 | 183,12 | 94,08 | 277,76 | 34,72 | 59,36 | 53,76 | 79,52 | 120,96 |
|  | 53,76 | 48,72 | 44,8 | 47,04 | 80,64 | 182,56 | 75,04 | 332,08 | 28 | 43,12 | 58,24 | 85,68 | 113,12 |
|  | 43,68 | 38,64 | 44,8 | 50,96 | 87,36 | 168 | 82,88 | 265,44 | 30,24 | 45,92 | 57,12 | 69,44 | 100,8 |
|  | 53,76 | 44,8 | 38,64 | 49,28 | 88,48 | 197,12 | 77,28 | 280 | 29,12 | 49,28 | 57,68 | 53,76 | 107,52 |
|  | 53,2 | 41,44 | 40,32 | 45,92 | 90,72 | 186,48 | 88,48 | 256,48 | 34,72 | 51,52 | 60,48 | 68,32 | 98,56 |
|  | 43,68 | 50,4 | 56 | 51,52 | 81,76 | 196 | 91,84 | 284,48 | 36,96 | 43,68 | 60,48 | 71,68 | 112,56 |
|  | 57,12 | 40,32 | 45,92 | 48,16 | 72,8 | 173,6 | 73,92 | 272,16 | 31,36 | 41,44 | 51,52 | 57,12 | 108,08 |
|  | 58,24 | 39,2 | 41,44 | 45,92 | 87,36 | 179,2 | 81,2 | 272,16 | 34,16 | 44,8 | 62,72 | 67,2 | 102,48 |
|  | 48,72 | 33,04 | 39,2 | 45,92 | 90,16 | 174,16 | 86,24 | 267,68 | 32,48 | 47,6 | 58,8 | 63,84 | 109,76 |
|  | 69,44 | 45,92 | 44,8 | 39,76 | 79,52 | 171,36 | 85,12 | 276,64 | 33,6 | 50,96 | 53,76 | 75,04 | 109,76 |
|  | 41,44 | 43,68 | 33,6 | 45,92 | 76,16 | 175,84 | 94,08 | 288,4 | 26,88 | 42,56 | 57,12 | 69,44 | 106,4 |
|  | 45,92 | 38,64 | 39,2 | 41,44 | 92,96 | 173,6 | 72,8 | 277,76 | 33,6 | 43,68 | 54,88 | 73,36 | 112 |
|  | 43,68 | 54,88 | 43,68 | 45,92 | 82,88 | 162,96 | 77,28 | 277,76 | 42,56 | 54,88 | 59,92 | 68,32 | 106,96 |
|  | 48,16 | 41,44 | 43,68 | 49,84 | 76,16 | 168 | 79,52 | 271,04 | 32,48 | 43,68 | 59,36 | 66,08 | 104,16 |
|  | 70,56 | 47,04 | 47,04 | 47,04 | 91,84 | 190,4 | 87,36 | 273,28 | 30,24 | 44,8 | 53,76 | 68,32 | 106,96 |
|  | 54,88 | 45,92 | 44,8 | 49,28 | 90,72 | 170,8 | 91,84 | 261,52 | 33,6 | 45,36 | 61,6 | 66,08 | 110,88 |
|  | 54,32 | 41,44 | 33,6 | 53,76 | 72,8 | 182 | 78,4 | 268,24 | 31,36 | 54,88 | 57,12 | 60,48 | 114,8 |
|  | 65,52 | 40,32 | 36,4 | 51,52 | 75,6 | 191,52 | 70,56 | 274,4 | 33,6 | 47,6 | 59,36 | 71,68 | 106,4 |
|  | 55,44 | 53,2 | 48,72 | 47,04 | 84 | 180,32 | 77,28 | 945,28 | 34,72 | 43,68 | 52,64 | 187,04 | 173,6 |
|  | 52,64 | 47,04 | 48,16 | 48,16 | 89,6 | 179,2 | 86,24 | 269,36 | 33,04 | 43,68 | 57,68 | 73,36 | 105,28 |
|  | 64,96 | 45,92 | 48,16 | 48,16 | 85,12 | 171,36 | 75,6 | 291,2 | 33,6 | 42,56 | 54,88 | 67,2 | 124,32 |
|  | 49,84 | 49,28 | 42,56 | 44,8 | 82,32 | 179,2 | 80,64 | 283,36 | 34,72 | 42,56 | 61,6 | 63,84 | 114,8 |
|  | 62,72 | 42,56 | 55,44 | 43,68 | 80,08 | 182,56 | 82,88 | 280,56 | 29,12 | 48,16 | 63,84 | 75,04 | 106,4 |
|  | 52,64 | 59,92 | 47,04 | 50,96 | 90,72 | 173,6 | 87,36 | 291,2 | 32,48 | 52,64 | 60,48 | 84 | 111,44 |
|  | 81,2 | 59,92 | 85,12 | 115,92 | 110,88 | 181,44 | 132,16 | 273,28 | 35,84 | 104,16 | 63,84 | 138,88 | 161,28 |
|  | 64,96 | 44,8 | 41,44 | 42 | 100,8 | 171,36 | 89,6 | 266,56 | 34,72 | 44,8 | 59,36 | 73,92 | 108,64 |
|  | 67,2 | 44,24 | 52,64 | 52,64 | 86,24 | 197,12 | 85,68 | 249,76 | 30,24 | 45,92 | 54,88 | 70,56 | 116,48 |
|  | 63,84 | 34,72 | 56 | 51,52 | 82,88 | 175,28 | 89,6 | 258,16 | 32,48 | 42,56 | 54,32 | 66,08 | 100,24 |
| CARDIAC | 67,2 | 40,32 | 48,16 | 53,76 | 95,2 | 171,36 | 84,56 | 282,24 | 33,6 | 52,08 | 53,2 | 71,68 | 110,32 |
|  | 56,56 | 49,28 | 43,68 | 52,08 | 80,64 | 194,88 | 76,16 | 280 | 30,24 | 42,56 | 56,56 | 62,72 | 100,8 |
|  | 49,28 | 50,96 | 40,32 | 53,2 | 81,76 | 187,6 | 82,88 | 276,64 | 26,88 | 46,48 | 58,24 | 64,96 | 106,4 |
|  | 58,24 | 50,96 | 39,2 | 47,6 | 94,64 | 202,72 | 84 | 273,28 | 31,36 | 47,04 | 57,12 | 62,72 | 104,72 |
|  | 73,36 | 33,04 | 40,32 | 48,72 | 94,08 | 181,44 | 85,12 | 282,24 | 30,8 | 44,24 | 58,24 | 77,84 | 114,24 |
|  | 79,52 | 54,88 | 52,64 | 50,96 | 81,2 | 210,56 | 69,44 | 271,04 | 28 | 51,52 | 61,6 | 76,16 | 117,6 |
|  | 47,04 | 35,84 | 38,08 | 59,36 | 79,52 | 189,28 | 72,24 | 276,08 | 26,88 | 45,92 | 61,04 | 66,08 | 107,52 |
|  | 63,84 | 33,6 | 36,96 | 57,12 | 95,76 | 182,56 | 72,8 | 268,8 | 42,56 | 42,56 | 58,24 | 75,04 | 108,64 |
|  | 60,48 | 45,92 | 47,04 | 51,52 | 93,52 | 176,96 | 89,6 | 281,12 | 31,36 | 49,28 | 53,76 | 61,6 | 103,04 |
|  | 75,04 | 38,08 | 40,32 | 57,12 | 81,76 | 179,2 | 87,36 | 286,72 | 30,24 | 44,8 | 54,88 | 66,64 | 107,52 |
|  | 39,2 | 30,24 | 41,44 | 43,12 | 81,76 | 173,04 | 78,4 | 285,04 | 32,48 | 40,32 | 52,08 | 67,2 | 96,32 |
|  | 52,64 | 45,92 | 40,32 | 53,76 | 78,96 | 181,44 | 80,08 | 287,84 | 30,24 | 45,92 | 54,88 | 60,48 | 108,64 |
|  | 44,8 | 43,12 | 45,92 | 44,24 | 73,92 | 182,56 | 82,88 | 283,36 | 31,36 | 45,92 | 60,48 | 68,32 | 110,88 |
|  | 43,68 | 45,92 | 53,76 | 44,8 | 75,6 | 187,6 | 85,12 | 268,8 | 36,96 | 53,76 | 61,6 | 68,32 | 109,76 |
|  | 52,64 | 42,56 | 45,92 | 44,8 | 85,12 | 179,2 | 90,72 | 268,8 | 35,84 | 47,04 | 56 | 67,2 | 108,64 |
|  | 63,84 | 33,6 | 39,76 | 50,4 | 84 | 185,92 | 75,04 | 277,76 | 32,48 | 42,56 | 57,12 | 68,32 | 110,88 |

**Table S2**. MFI of patients with chronic Chagas cardiomyopathy infected with different DTU´s and control

| GROUP | IL-12 | INF-γ | IL-17 | IL-2 | IL-10 | IL-9 | IL-22 | IL-6 | IL-13 | IL-4 | IL-5 | IL-1β | TNF-α |
| --- | --- | --- | --- | --- | --- | --- | --- | --- | --- | --- | --- | --- | --- |
| Tc I | 255,36 | 266,56 | 234,64 | 198,24 | 434,56 | 781,76 | 339,92 | 1183,84 | 184,8 | 204,96 | 273,28 | 278,88 | 490,56 |
|  | 218,4 | 271,04 | 220,08 | 316,4 | 382,48 | 819,84 | 461,44 | 1350,72 | 191,52 | 295,12 | 308 | 356,16 | 501,76 |
|  | 166,88 | 166,88 | 186,48 | 166,32 | 295,68 | 732,48 | 329,28 | 1148 | 126 | 183,68 | 227,36 | 260,96 | 442,96 |
|  | 192,64 | 208,32 | 185,92 | 192,64 | 332,08 | 728 | 357,28 | 1211,28 | 161,28 | 212,8 | 226,24 | 286,72 | 456,96 |
|  | 164,64 | 143,36 | 140,56 | 183,68 | 326,48 | 729,12 | 342,72 | 1177,12 | 136,64 | 188,72 | 248,64 | 285,04 | 437,92 |
|  | 210,56 | 187,04 | 216,16 | 244,16 | 328,16 | 704,48 | 355,04 | 1233,68 | 142,24 | 225,12 | 199,36 | 305,76 | 441,84 |
|  | 237,44 | 180,32 | 198,8 | 231,28 | 363,44 | 954,24 | 370,72 | 1175,44 | 162,4 | 209,44 | 253,68 | 240,8 | 466,48 |
|  | 202,72 | 197,12 | 182,56 | 173,6 | 336 | 717,36 | 312,48 | 1178,8 | 162,4 | 198,24 | 218,4 | 303,52 | 464,8 |
|  | 407,12 | 225,12 | 213,92 | 221,76 | 378,56 | 792,4 | 310,24 | 1220,8 | 123,76 | 181,44 | 230,72 | 271,04 | 446,88 |
|  | 212,24 | 176,96 | 246,4 | 217,84 | 388,64 | 760,48 | 351,68 | 1256,64 | 168,56 | 234,08 | 269,92 | 292,32 | 456,96 |
|  | 199,36 | 135,52 | 153,44 | 224 | 317,52 | 706,16 | 278,88 | 1125,6 | 146,16 | 190,4 | 227,36 | 268,8 | 399,84 |
|  | 540,4 | 423,36 | 299,04 | 348,88 | 516,32 | 945,28 | 547,12 | 1201,76 | 151,76 | 271,04 | 272,72 | 365,12 | 530,32 |
|  | 295,68 | 174,72 | 173,6 | 193,2 | 338,24 | 729,68 | 357,28 | 1164,8 | 145,6 | 198,24 | 215,04 | 257,6 | 436,8 |
|  | 217,28 | 199,36 | 193,2 | 212,8 | 409,92 | 826,56 | 390,88 | 1277,92 | 156,8 | 193,76 | 227,36 | 321,44 | 496,16 |
|  | 620,48 | 478,8 | 255,92 | 338,24 | 557,2 | 778,4 | 402,08 | 1289,68 | 156,8 | 282,8 | 286,72 | 304,64 | 502,88 |
|  | 353,36 | 241,36 | 241,92 | 233,52 | 379,68 | 753,76 | 352,8 | 1197,84 | 175,84 | 220,64 | 260,96 | 273,28 | 461,44 |
|  | 211,12 | 202,16 | 190,4 | 234,08 | 339,36 | 724,64 | 349,44 | 1168,72 | 151,2 | 209,44 | 250,88 | 269,92 | 445,76 |
|  | 205,52 | 179,2 | 194,88 | 234,64 | 397,6 | 775,04 | 355,6 | 1168,72 | 171,36 | 203,84 | 258,16 | 287,84 | 490,56 |
|  | 234,08 | 194,88 | 164,08 | 204,96 | 324,8 | 713,44 | 343,84 | 1156,96 | 154,56 | 203,84 | 232,4 | 272,72 | 419,44 |
|  | 154,56 | 194,88 | 164,64 | 170,8 | 357,84 | 785,68 | 355,04 | 1190,56 | 149,52 | 189,28 | 247,52 | 263,2 | 445,76 |
| Tc II | 238 | 216,16 | 184,8 | 210,56 | 376,32 | 718,48 | 400,4 | 1120 | 156,8 | 198,24 | 255,36 | 287,84 | 442,4 |
|  | 210,56 | 189,28 | 222,88 | 236,88 | 355,04 | 770,56 | 421,12 | 1152,48 | 155,68 | 177,52 | 251,44 | 285,04 | 467,6 |
|  | 168 | 192,64 | 168 | 218,4 | 291,2 | 740,32 | 283,36 | 1162,56 | 151,2 | 191,52 | 232,4 | 265,44 | 437,36 |
|  | 211,68 | 188,16 | 210,56 | 221,76 | 356,16 | 749,84 | 411,04 | 1189,44 | 157,92 | 208,88 | 240,8 | 293,44 | 463,68 |
|  | 259,84 | 194,88 | 213,36 | 276,64 | 407,68 | 721,28 | 374,08 | 1246,56 | 185,92 | 230,72 | 293,44 | 316,96 | 468,16 |
|  | 228,48 | 182 | 207,76 | 225,12 | 344,4 | 826,56 | 366,24 | 1205,68 | 178,08 | 226,8 | 248,64 | 302,4 | 506,24 |
|  | 7,84 | 173,6 | 169,12 | 77,28 | 369,6 | 823,2 | 134,96 | 1142,96 | 130,48 | 124,32 | 222,32 | 278,88 | 454,72 |
|  | 246,4 | 172,48 | 203,84 | 185,92 | 293,44 | 732,48 | 283,36 | 1171,52 | 208,88 | 237,44 | 203,84 | 254,24 | 450,24 |
|  | 266,56 | 182,56 | 208,32 | 231,84 | 366,24 | 744,8 | 387,52 | 1169,28 | 176,96 | 215,04 | 248,64 | 327,04 | 456,96 |
|  | 240,24 | 176,96 | 208,32 | 246,4 | 404,88 | 736,96 | 365,12 | 1148 | 169,12 | 212,8 | 259,84 | 300,16 | 451,36 |
|  | 278,88 | 229,6 | 281,12 | 276,64 | 446,88 | 764,96 | 401,52 | 1296,4 | 183,12 | 205,52 | 284,48 | 352,8 | 462,56 |
|  | 118,72 | 217,28 | 208,88 | 220,64 | 342,72 | 760,48 | 490 | 1252,16 | 165,76 | 199,36 | 258,72 | 318,08 | 470,4 |
|  | 219,52 | 273,28 | 260,96 | 190,4 | 348,32 | 799,68 | 747,04 | 1239,84 | 153,44 | 236,88 | 277,76 | 268,24 | 479,92 |
|  | 1393,28 | 1251,6 | 286,16 | 289,52 | 696,64 | 1100,96 | 953,12 | 1593,2 | 225,12 | 446,32 | 483,28 | 525,28 | 553,84 |
|  | 243,04 | 248,64 | 252,56 | 288,96 | 331,52 | 923,44 | 857,36 | 1255,52 | 182,56 | 246,4 | 225,12 | 249,2 | 464,24 |
|  | 215,04 | 206,64 | 206,08 | 291,2 | 347,2 | 884,8 | 723,52 | 1281,28 | 240,8 | 208,32 | 288,96 | 312,48 | 477,12 |
|  | 225,68 | 235,2 | 189,28 | 224 | 378 | 919,52 | 698,32 | 1202,32 | 134,4 | 197,12 | 254,24 | 322,56 | 445,2 |
|  | 245,28 | 197,68 | 249,76 | 175,84 | 384,16 | 764,96 | 745,92 | 1146,88 | 164,64 | 239,68 | 267,68 | 249,2 | 517,44 |
|  | 208,88 | 142,24 | 235,2 | 227,36 | 390,88 | 753,2 | 642,88 | 1970,08 | 151,2 | 239,68 | 225,12 | 377,44 | 478,24 |
|  | 274,4 | 169,68 | 204,96 | 277,76 | 381,92 | 943,6 | 682,64 | 1504,72 | 164,64 | 226,8 | 285,04 | 371,28 | 490,56 |
| MIX : Tc I + Tc II | 194,88 | 177,52 | 172,48 | 210,56 | 320,32 | 931,84 | 786,8 | 1233,68 | 165,76 | 199,36 | 246,4 | 318,08 | 471,52 |
|  | 309,12 | 251,44 | 256,48 | 320,32 | 496,16 | 834,4 | 684,32 | 1232 | 163,52 | 291,2 | 281,12 | 302,4 | 478,24 |
|  | 206,08 | 188,16 | 181,44 | 263,76 | 369,6 | 938,56 | 672 | 1151,36 | 190,4 | 183,68 | 236,32 | 290,08 | 423,92 |
|  | 212,8 | 198,24 | 234,08 | 245,28 | 459,2 | 796,32 | 726,88 | 1207,92 | 154,56 | 212,8 | 255,36 | 357,84 | 459,2 |
|  | 187,04 | 182 | 220,64 | 188,16 | 318,64 | 1012,48 | 768,88 | 1196,72 | 158,48 | 157,92 | 231,84 | 254,8 | 446,88 |
|  | 208,32 | 193,76 | 226,24 | 217,28 | 358,4 | 875,28 | 657,44 | 1167,04 | 160,16 | 241,92 | 238,56 | 228,48 | 465,92 |
|  | 206,08 | 180,32 | 161,84 | 230,72 | 375,2 | 847,84 | 542,08 | 1153,6 | 135,52 | 197,12 | 236,32 | 249,2 | 476 |
|  | 208,32 | 155,12 | 204,4 | 221,76 | 447,44 | 911,12 | 764,96 | 1222,48 | 173,6 | 277,2 | 276,64 | 309,12 | 539,84 |
|  | 173,6 | 192,64 | 187,04 | 250,32 | 328,72 | 855,68 | 717,36 | 1216,32 | 144,48 | 201,04 | 223,44 | 302,4 | 514,08 |
|  | 212,24 | 211,68 | 200,48 | 227,36 | 409,92 | 784 | 738,08 | 1266,72 | 171,36 | 235,2 | 275,52 | 292,88 | 487,2 |
|  | 223,44 | 220,64 | 165,2 | 216,72 | 359,52 | 846,72 | 619,36 | 1198,4 | 150,08 | 201,6 | 280 | 318,08 | 445,76 |
|  | 180,32 | 166,32 | 229,6 | 209,44 | 353,92 | 892,64 | 611,52 | 1160,32 | 182,56 | 187,04 | 247,52 | 315,28 | 462,56 |
|  | 192,64 | 194,88 | 154,56 | 233,52 | 313,6 | 871,36 | 628,32 | 1193,92 | 151,2 | 216,16 | 266,56 | 266,56 | 463,12 |
|  | 266,56 | 201,6 | 228,48 | 297,92 | 392 | 1034,88 | 969,92 | 1144,64 | 172,48 | 292,32 | 267,68 | 331,52 | 455,84 |
|  | 229,6 | 198,24 | 202,72 | 218,4 | 346,08 | 776,16 | 582,4 | 1136,24 | 137,2 | 181,44 | 244,16 | 302,4 | 436,24 |
| CONTROL | 235,2 | 230,16 | 225,12 | 280 | 409,36 | 776,16 | 640,08 | 1178,24 | 198,24 | 241,92 | 280,56 | 314,72 | 495,04 |
|  | 722,4 | 463,68 | 282,8 | 263,2 | 591,92 | 956,48 | 857,36 | 1446,48 | 141,12 | 216,72 | 358,96 | 336 | 483,28 |
|  | 230,72 | 164,64 | 237,44 | 250,88 | 358,96 | 859,04 | 551,6 | 1253,28 | 217,84 | 206,64 | 274,96 | 306,32 | 472,64 |
|  | 532 | 325,92 | 220,64 | 264,32 | 422,24 | 834,4 | 716,8 | 1167,04 | 174,72 | 211,68 | 262,64 | 343,84 | 498,4 |
|  | 518,56 | 333,76 | 226,8 | 247,52 | 417,76 | 828,8 | 625,52 | 1193,36 | 184,8 | 217,28 | 250,88 | 255,36 | 492,8 |
|  | 515,76 | 310,8 | 193,76 | 199,36 | 398,72 | 877,52 | 397,6 | 1168,16 | 156,8 | 211,68 | 238 | 264,88 | 445,76 |
|  | 575,12 | 312,48 | 197,12 | 190,4 | 353,92 | 828,24 | 547,68 | 1200,64 | 160,16 | 208,32 | 256,48 | 259,28 | 496,16 |
|  | 589,12 | 328,16 | 211,68 | 206,08 | 383,6 | 812 | 747,04 | 1243,2 | 141,68 | 193,2 | 256,48 | 273,28 | 497,28 |
|  | 646,24 | 341,04 | 216,16 | 219,52 | 370,72 | 852,88 | 798,56 | 1193,92 | 146,72 | 218,4 | 222,88 | 224 | 496,72 |
|  | 661,92 | 294 | 189,84 | 193,2 | 386,4 | 723,52 | 799,68 | 1200,64 | 152,32 | 182,56 | 241,36 | 306,88 | 490,56 |
|  | 510,16 | 346,08 | 185,92 | 207,76 | 347,2 | 784 | 646,24 | 1260 | 138,88 | 141,68 | 236,32 | 281,12 | 474,88 |
|  | 495,04 | 358,96 | 184,8 | 215,04 | 339,36 | 761,6 | 688,8 | 1165,92 | 160,16 | 243,04 | 256,48 | 285,6 | 458,64 |

**Table S3.** Kaiser-Meyer**-**Olkin and Barlett´s Test for with and without chronic cardiomyopathy chagasic patients and control.

**KMO and Bartlett´s Test**

| Kaiser-Meyer-Olkin Measure of Sampling | | 0,649 |
| --- | --- | --- |
| Bartlett´s Test of Sphericity | Approx. Chi-cuadrado | 585,314 |
|  | df | 78 |
|  | Sig. | 0,000 |

**Table S4**. Communalities for with and without chronic cardiomyopathy chagasic patients and control.

**Communalities ^a.^**

|  | Initial | Extraction ^b..^ |
| --- | --- | --- |
| IFN | 1,000 | 0,990 |
| IL12 | 1,000 | 0,990 |
| IL1 | 1,000 | 0,920 |
| IL6 | 1,000 | 0,915 |
| IL9 | 1,000 | 0,898 |
| IL4 | 1,000 | 0,864 |
| IL2 | 1,000 | 0,815 |
| IL17 | 1,000 | 0,761 |
| IL22 | 1,000 | 0,753 |
| IL13 | 1,000 | 0,688 |
| TNF | 1,000 | 0,679 |
| IL5 | 1,000 | 0,614 |
| IL10 | 1,000 | 0,571 |

1. Extraction Method: APC
2. Extraction: Is the proportion of each variable variance explains by the factors. Higher levels are been represented in the space

**Table S5**. Total Variance Explanied for with and without chronic cardiomyopathy chagasic patients and control.

| Component | Initial Eigenvalues | | | Extraction Sums of Squared Loadings | | | Rotation Sums of Squared Loading | | |
| --- | --- | --- | --- | --- | --- | --- | --- | --- | --- |
|  | Total | % of Variance | %  Cumulative | Total | % of Variance | % Cumulative | Total | %of Variance | % Cumulative |
| IFN | 4,400 | 33,845 | 33,845 | 4,400 | 33,845 | 33,845 | 3,710 | 28,538 | 28,538 |
| IL12 | 2,072 | 15,938 | 49,782 | 2,072 | 15,938 | 49,782 | 2,005 | 15,422 | 43,960 |
| IL1 | 1,732 | 13,325 | 63,107 | 1,732 | 13,325 | 63,107 | 1,991 | 15,314 | 59,274 |
| IL6 | 1,205 | 9,273 | 72,380 | 1,205 | 9,273 | 72,380 | 1,576 | 12,123 | 71,398 |
| IL9 | 1,049 | 8,073 | 80,452 | 1,049 | 8,073 | 80,452 | 1,177 | 9,055 | 80,452 |
| IL4 | ,685 | 5,267 | 85,719 |  |  |  |  |  |  |
| IL2 | ,551 | 4,242 | 89,961 |  |  |  |  |  |  |
| IL17 | ,487 | 3,747 | 93,708 |  |  |  |  |  |  |
| IL22 | ,314 | 2,415 | 96,123 |  |  |  |  |  |  |
| IL13 | ,288 | 2,216 | 98,339 |  |  |  |  |  |  |
| TNF | ,151 | 1,165 | 99,504 |  |  |  |  |  |  |
| IL5 | ,052 | ,396 | 99,900 |  |  |  |  |  |  |
| IL10 | ,013 | ,100 | 100,000 |  |  |  |  |  |  |

**Table S6.** Kaiser-Meyer**-**Olkin and Barlett´s Test for patients with chronic Chagas cardiomyopathy infected with different DTU´s and control

**KMO and Bartlett´s Test**

| Kaiser-Meyer-Olkin Measure of Sampling | | 0,807 |
| --- | --- | --- |
| Bartlett´s Test of Sphericity | Approx. Chi-cuadrado | 579,955 |
|  | df | 78 |
|  | Sig. | 0,000 |

**Table S7.** Communalities for patients with chronic Chagas cardiomyopathy infected with different DTU´s and control

**Communalities ^a.^**

|  | Initial | Extraction ^b..^ |
| --- | --- | --- |
| IFN | 1 | 0,882 |
| IL12 | 1 | 0,822 |
| IL22 | 1 | 0,817 |
| IL10 | 1 | 0,805 |
| IL9 | 1 | 0,775 |
| IL5 | 1 | 0,771 |
| IL4 | 1 | 0,716 |
| IL1 | 1 | 0,643 |
| IL2 | 1 | 0,638 |
| IL13 | 1 | 0,628 |
| IL17 | 1 | 0,552 |
| TNF | 1 | 0,506 |
| IL6 | 1 | 0,65 |

1. Extraction Method: APC
2. Extraction: Is the proportion of each variable variance explains by the factors. Higher levels are been represented in the space

**Table S8.** Total Variance Explanied for patients with chronic Chagas cardiomyopathy infected with different DTU´s and control

| Component | Initial Eigenvalues | | | Extraction Sums of Squared Loadings | | | Rotation Sums of Squared Loading | | |
| --- | --- | --- | --- | --- | --- | --- | --- | --- | --- |
|  | Total | % of Variance | % | Total | % of Variance | % Cumulative | Total | % of Variance | % Cumulative |
|  |  |  | Cumulative |  |  |  |  |  |  |
| IFN | 6,577 | 50,592 | 50,592 | 6,577 | 50,592 | 50,592 | 4,325 | 33,270 | 33,270 |
| IL12 | 1,188 | 9,141 | 59,733 | 1,188 | 9,141 | 59,733 | 2,591 | 19,927 | 53,198 |
| IL1 | 1,138 | 8,752 | 68,485 | 1,138 | 8,752 | 68,485 | 1,987 | 15,287 | 68,485 |
| IL6 | 0,902 | 6,937 | 75,421 |  |  |  |  |  |  |
| IL9 | 0,734 | 5,643 | 81,064 |  |  |  |  |  |  |
| IL4 | 0,627 | 4,822 | 85,887 |  |  |  |  |  |  |
| IL2 | 0,444 | 3,412 | 89,299 |  |  |  |  |  |  |
| IL17 | 0,422 | 3,244 | 92,543 |  |  |  |  |  |  |
| IL22 | 0,311 | 2,396 | 94,939 |  |  |  |  |  |  |
| IL13 | 0,285 | 2,189 | 97,128 |  |  |  |  |  |  |
| TNF | 0,203 | 1,559 | 98,687 |  |  |  |  |  |  |
| IL5 | 0,123 | 0,949 | 99,636 |  |  |  |  |  |  |
| IL10 | 0,047 | 0,364 | 100,000 |  |  |  |  |  |  |

**Table S9.** Classification Result for discriminant analyze for with and without chronic cardiomyopathy chagasic patients and control.

| **Classification Results^a^** | | | | | | |
| --- | --- | --- | --- | --- | --- | --- |
|  |  |  | Predicted Group Membership | | | Total |
|  |  |  | CONTROL | NON-CARD | CARD |  |
| Original | Count | CONTROL | 5 | 4 | 0 | 9 |
|  |  | NON-CARD | 11 | 21 | 6 | 38 |
|  |  | CARD | 0 | 0 | 16 | 16 |
|  | % | CONTROL | 55,6 | 44,4 | 0 | 100 |
|  |  | NON-CARD | 28,9 | 55,3 | 15,8 | 100 |
|  |  | CARD | 0 | 0 | 100 | 100 |
| a. 66.7% OF Original grouped cases correctly classify. | | | | | | |

**Table S10.** Classification Result for discriminant analyze for patients with chronic Chagas cardiomyopathy infected with different DTU´s and control

| **Classification Results^a^** | | | | | | | |
| --- | --- | --- | --- | --- | --- | --- | --- |
|  |  |  | Predicted Group Membership | | | | Total |
|  |  |  | Tc I | Tc II | Mix Tc I/TcII | Control |  |
| Original | Count | Tc I | 19 | 1 | 0 | 0 | 20 |
|  |  | Tc II | 10 | 3 | 7 | 0 | 20 |
|  |  | Mixed TcI/TcII | 0 | 2 | 13 | 0 | 15 |
|  |  | Control | 0 | 1 | 1 | 10 | 12 |
|  | % | Tc I | 95 | 5 | 0 | 0 | 100 |
|  |  | Tc II | 50 | 15 | 35 | 0 | 100 |
|  |  | Mixed TcI/TcII | 0 | 13,3 | 86,7 | 0 | 100 |
|  |  | Control | 0 | 8,3 | 8,3 | 83,3 | 100 |
| a. 67.2% of Original grouped cases correctly classify. | | | | | | | |
